# A GaSe/Si-based vertical 2D/3D heterojunction for high-performance self-driven photodetectors

**DOI:** 10.1039/d1na00659b

**Published:** 2021-12-02

**Authors:** Sahin Sorifi, Shuchi Kaushik, Rajendra Singh

**Affiliations:** Department of Physics, Indian Institute of Technology Delhi New Delhi 110016 India rsingh@physics.iitd.ac.in; Nanoscale Research Facility, Indian Institute of Technology Delhi New Delhi 110016 India

## Abstract

We report on the fabrication of a vertical 2D/3D heterojunction diode between gallium selenide (GaSe) and silicon (Si), and describe its photoresponse properties. Kelvin probe force microscopy (KPFM) has been employed to investigate the surface potentials of the GaSe/Si heterostructure, leading to the evaluation of the value of the conduction band offset at the heterostructure interface. The current–voltage measurements on the heterojunction device display a diode-like nature. This diode-like nature is attributed to the type-II band alignment that exists at the p–n interface. The key parameters of a photodetector, such as photoresponsivity, detectivity, and external quantum efficiency, have been calculated for the fabricated device and compared with those of other similar devices. The photodetection measurements of the GaSe/Si heterojunction diode show excellent performance of the device, with high photoresponsivity, detectivity, and EQE values of ∼2.8 × 10^3^ A W^−1^, 6.2 × 10^12^ Jones, and 6011, respectively, at a biasing of −5 V. Even at zero biasing, a high photoresponsivity of 6 A W^−1^ was obtained, making it a self-powered device. Therefore, the GaSe/Si self-driven heterojunction diode has promising potential in the field of efficient optoelectronic devices.

## Abstract 2

We have devised a vertical self-powered GaSe/Si based 2D/3D p–n heterojunction device for a high-performance photodetection application.

## Graphical abstract

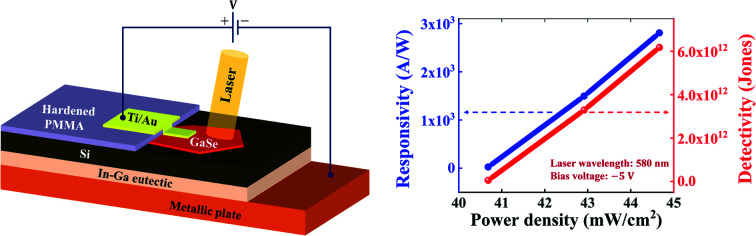

## Introduction

Dimensionality is one of the crucial parameters to define the property of a material. The same material can exhibit a dramatic change in its behavior depending on whether it is arranged in a zero-dimensional (0D),^1^ one-dimensional (1D),^2^ two-dimensional (2D),^3^ or three-dimensional (3D)^4^ crystal structure. Ever since the successful isolation of a single layer of graphite through mechanical exfoliation in 2004 by Novoselov and Geim,^5^ two-dimensional layered materials (TDLMs) such as transition metal dichalcogenides (TMDCs),^6,7^ and transition metal monochalcogenides (TMMCs)^8^ garnered tremendous attention of the scientific community. These materials overcame the zero band-gap limitation of graphene and emerged to be revolutionary in the field of optoelectronics and microelectronics with their highly promising properties, such as layer-dependent bandgaps, efficient light absorption, flexibility, and alluring electrical tunability.^9,10^ The weak van der Waals (vdW) forces existing between the different layers of TDLMs help to cleave them in layers; moreover, the absence of dangling bonds in these materials facilitates the integration of different TDLMs to realize van der Waals heterostructures (vdWHs).^11–13^ These ultrathin vdWHs, such as GaSe–MoS_2_,^14^ GaSe–InSe,^15^ GaSe–WS_2_,^16^ GaS–GaSe,^17^ SnSe_2_–MoSe_2_,^18^ and GaSe–MoSe_2_,^19^ have been fabricated for potential applications in several optoelectronics devices for their high sensitivity, high speed, and wide range of photoresponse properties. Beyond these 2D heterostructures, the TDLMs can also be integrated with bulk 3D semiconducting materials without any problem of crystal lattice mismatch. In these mixed-dimensional vdWHs, the advantageous properties of both 2D and 3D materials can be utilized in a single device.^20–23^ In the recent past, several research groups implemented this idea of utilizing the complementary properties of different dimensions and integrated the 2D layered materials with several bulk materials, such as SiC,^24^ GaN,^25^ and Si.^26,27^ In particular, the 2D/3D heterojunctions between 2D layered materials and 3D Si have shown immense potential for large-scale practical applications, such as highly efficient solar cells^28,29^ and photodetectors.^30,31^ To date, most of the reported 2D/3D p–n heterojunction-based photodetectors have been limited to the combination of Si with graphene^32^ or with TMDCs such as MoS_2_^26,33^ and most recently a few have involved combining graphene with GaN.^34,35^ There are very few reports available on p–n heterojunctions based on Si and TMMCs such as InSe and GaSe. In the recent past, several research groups developed GaSe-based photodetectors which were found to be very promising in terms of their detectivity, photoresponsivity, speed, and noise equivalent power (NEP).^36,37^ Yan *et al.*^38^ provided a brief account of some high-performance selenide-based vdW heterojunction photodetectors, such as SnSe/Si^39^ and InSe/Si.^40^ However, the photoresponse properties still require some improvements to find potential for commercial applications. One of the most effective ways to do this is to integrate this intrinsic p-type GaSe with the state-of-the-art silicon-based technology.

GaSe is a III–VI semiconducting material with a direct bandgap of ∼2 eV in the multi-layer form and an indirect bandgap of ∼4 eV when prepared in a monolayer form.^41^ Each layer of this layered material comprises a respective sequence of four atoms, Se–Ga–Ga–Se, and a weak van der Waals force exists between the different layers, enabling it to be exfoliated in layers *via* mechanical exfoliation.^42^

In the present work, we report about the fabrication and characterization of a vertically stacked exfoliated GaSe/Si-based 2D/3D p–n heterojunction. A type-II band alignment is present at the p-GaSe/n-Si interface which modulates and tunes the optoelectronic properties of GaSe effectively. The Kelvin probe force microscopy (KPFM) investigation confirms that a difference of 146 mV in the surface potential values exists between Si and GaSe; this results in an interfacial barrier, which causes quick separation of the charge carriers. The heterojunction shows a rectifying nature, with a high current rectification ratio of about 10^3^ evaluated at ±5 V. Photoresponse measurements on the fabricated device indicate excellent performance, with high photoresponsivity, detectivity, and EQE values of ∼2.8 × 10^3^ A W^−1^, 6.2 × 10^12^ Jones, and 6011, respectively. Moreover, a strong photoresponse of 6 A W^−1^ has also been observed even at zero biasing. The inbuilt potential at the heterostructure enables the device to work even without power, making it a self-driven photodetector. Therefore, this GaSe/Si-based p–n vertical heterojunction holds great potential to enrich the field of electronic and optoelectronic devices.

## Experimental section

To fabricate the vertical p-GaSe/n-Si heterojunction, an n-type silicon (100) wafer with a thickness of ∼400 μm and p-type GaSe (purchased from 2D Semiconductors, USA) were used. Firstly, the n-type silicon wafer was given an ultrasonic bath to remove all the contamination from its surface. It was treated successively in acetone, isopropyl alcohol, and de-ionized (DI) water for 5 minutes each at room temperature. Metallic marks were fabricated on the cleaned Si substrate, with the help of electron beam lithography (EBL) and electron beam evaporation, so that the position of the micro-sized GaSe flakes could be easily traced. A small amount of the bulk GaSe crystal was taken on scotch tape, and it was uniformly distributed all over the tape. The tape was then slightly pressed onto the cleaned marked n-Si substrate and peeled off. This mechanically exfoliated GaSe possesses better crystallinity with fewer defects than chemically produced GaSe.

The thicknesses of the transferred GaSe films were precisely measured by employing atomic force microscopy (AFM) in tapping mode (model: Dimension ICON from Bruker), and the surface potential measurements were performed using multi-modal KPFM (model: Dimension ICON from Bruker) in tapping mode. Raman measurements were carried out with 514 nm laser excitation in a Micro-Raman system at a power of 40 mW to confirm the successful transfer of GaSe flakes on Si (model: Lab RAM HR evolution from Horiba). The chemical compositions at the p–n heterojunction were estimated using energy dispersive X-ray spectroscopy (EDS) (Hitachi tabletop microscope, TM 3000).

**Fig. 1:**
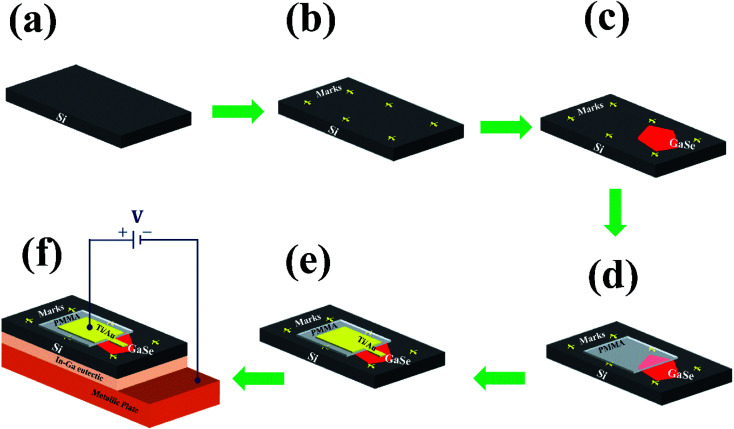
Complete device fabrication process. (a) Cleaned Si (100) substrate; (b) fabrication of plus marks; (c) transfer of GaSe flake on the Si substrate using mechanical exfoliation; (d) PMMA hardened near the edge of the GaSe flake to insulate the electrode; (e) formation of the electrode on the GaSe flake; (f) schematic of the final device.

The device was fabricated using EBL (model: eLine plus Raith GmbH). To insulate the electrode over GaSe from Si, a 300 nm thick PMMA (polymethyl methacrylate) layer with a molecular weight of 950k was hardened at the GaSe flake in such a way that it partially lies on the flake. The PMMA was exposed to an optimized high dose with an energy density of 7000 μC cm^−2^ to harden it and later developed in acetone followed by rinsing in isopropyl alcohol. One more step of EBL was performed to pattern the contact electrode on the GaSe flake. Ti (30 nm) with a capping layer of Au (60 nm) was deposited on the electrode with the help of e-beam evaporation, and the lift-off process was performed in acetone. The complete process of fabricating the device was carried out inside class 100 clean rooms, avoiding any unwanted contamination effects. The complete process of the device fabrication is shown in Fig. 1. A DC probe station (model: EverBeing-EB6) coupled with a semiconductor characterization system (Keithley: SCS-4200), placed inside a black case, was employed to carry out the electrical characterization of the fabricated device. A 75 W xenon lamp was used for the photodetection measurements. The power density of the incident light (580 nm laser) was measured with the help of a silicon power detector from Thorlabs (model: S-130 VC) and a power meter (model: PM-100D).

## Results and discussion

**Fig. 2:**
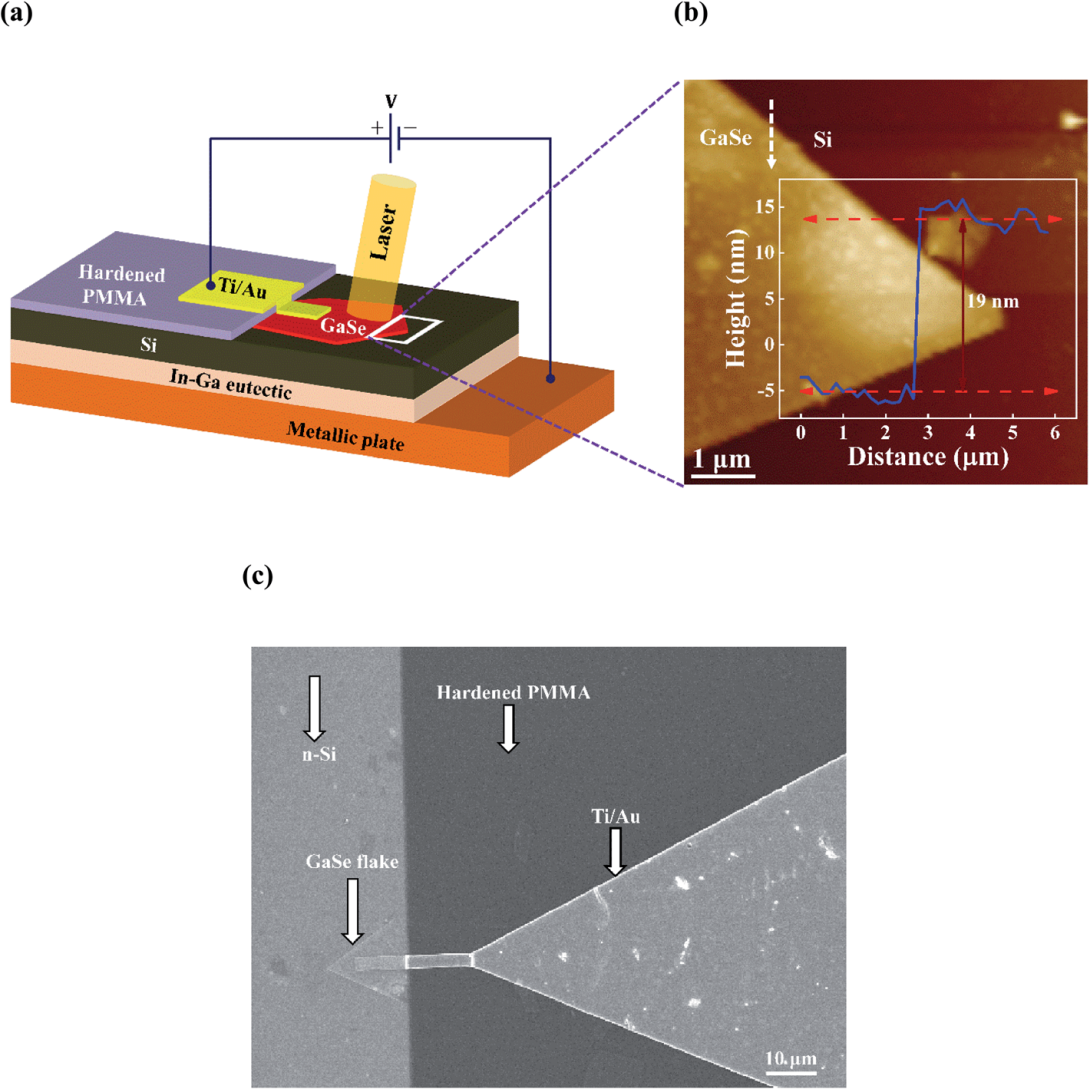
(a) A schematic of the top view of the GaSe/Si p–n heterojunction photodetector. (b) AFM micrograph of the GaSe/Si heterojunction, where the inset graph represents the height profile along the white dashed arrow. (c) FESEM image of the as-fabricated device.

The mechanically exfoliated GaSe thin flakes, transferred onto the Si substrate, were observed under a field emission scanning electron microscope (FESEM), and suitable flakes were chosen for device fabrication. Fig. 2a presents a schematic of the fabricated device. The AFM image of the vertical p–n heterojunction formed between the p-type GaSe thin flake and the n-type Si within the rectangular region, as indicated in Fig. 2a, is shown in Fig. 2b. AFM in tapping mode was employed to characterize the surface morphology of the p-GaSe/n-Si heterojunction (Fig. 2b). The inset in Fig. 2b presents the height profile corresponding to the drawn arrow line, and it shows that the thickness of the GaSe flake is approximately 19 nm. The FESEM image of the as-fabricated device is shown in Fig. 2c.

**Fig. 3:**
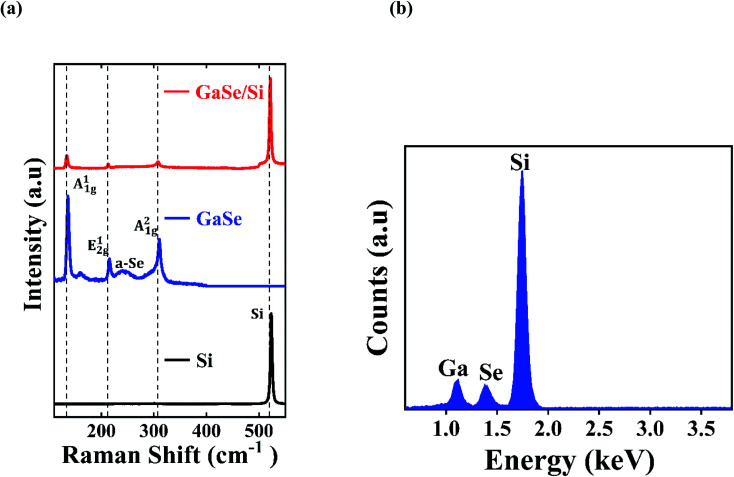
(a) Raman spectra of isolated Si, GaSe, and their overlapped region. (b) EDS spectrum of the GaSe/Si p–n heterojunction.

The existence of the exfoliated GaSe flake on the Si substrate was confirmed using Raman spectroscopic measurement with a 514 nm laser excitation (of 40 mW power). The Raman spectrum of the sample at the pristine GaSe, Si, and GaSe/Si overlapped region sites is shown in Fig. 3a. As can be observed from Fig. 3a, GaSe gives three dominant peaks, positioned at 133.0 cm^−1^ (A^1^_1g_), 307.0 cm^−1^ (A^2^_1g_), and 212.5 cm^−1^ (E^1^_2g_). Among these three Raman-active modes, E^1^_2g_ is an in-plane vibration mode and A^1^_1g_ and A^2^_1g_ are out-of-plane vibration modes. These Raman modes are in complete agreement with the previously reported Raman spectrum of GaSe.^8^ In addition to these three main peaks, a broad feature, positioned at 249.0 cm^−1^, can be seen in the Raman spectrum. The reason for the appearance of this feature lies in the formation of amorphous selenium (a-Se) in GaSe from the inter-chain bond stretching of the disordered selenium.^43^ In the Raman spectrum of Si, a high-intensity peak is located at 521 cm^−1^. All the characteristic peaks of both GaSe and Si are observed in the GaSe/Si overlapped region without any noticeable change in their peak positions, as shown in Fig. 3a. As the Raman peaks of GaSe are generated from the thin GaSe nanoflakes, their intensities are much lower compared to the Raman peak of bulk Si. Moreover, according to Doan *et al.*,^44^ the interfacial coupling between the two materials of the heterojunction may also contribute to the reduction of intensities of the GaSe peaks. The energy dispersive X-ray spectrum (EDS), derived at the GaSe/Si heterojunction, also confirms the existence of GaSe and Si in the heterostructure. Fig. 3b clearly shows the signals from the elements Ga, Se, and Si. The peaks for Ga and Se appear at almost the same height, indicating that the stoichiometric amounts of Ga and Se are in the ratio of 1 : 1.

**Fig. 4:**
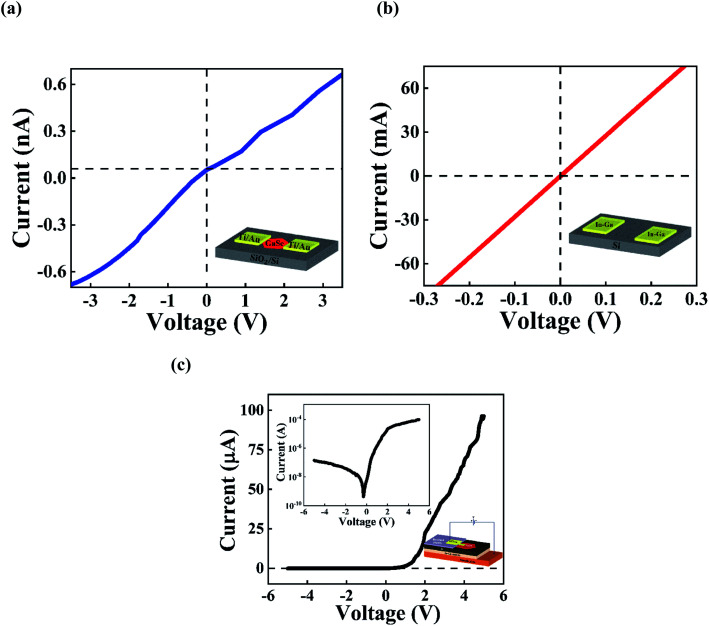
*I*–*V* measurements on (a) p-GaSe and (b) n-Si in dark conditions using the Ti/Au and In–Ga eutectic electrodes, respectively. The insets present the schematics of the arrangements for carrying out the *I*–*V* measurements. (c) *I*–*V* characteristics of the p-GaSe/n-Si heterojunction diode under dark conditions at room temperature, showing a clear current rectification.

Before carrying out the photodetection measurements on the GaSe/Si vertical p–n heterojunction, the current–voltage (*I*–*V*) characteristics of the heterostructure were examined in dark conditions at room temperature. The *I*–*V* curve from the heterostructure in Fig. 4c shows a very clear diode-like nature with a high current rectification ratio. Prior to the fabrication of the heterostructure, we conducted a separate study on the ohmic contact formations on n-Si and p-GaSe. Eutectic In–Ga provided perfect ohmic behavior on n-Si, and nearly ohmic behavior was observed in the case of p-GaSe with Ti(30 nm)/Au(60 nm) as metal contacts. The linear *I*–*V* characteristics on p-GaSe are shown in Fig. 4a and those for n-Si are shown in Fig. 4b. The lower dark current drawn from the p-GaSe, as compared to the n-Si, is attributed to the lower hall mobility and higher resistivity of the p-type GaSe layered structure than of the bulk n-Si.^45^ Since both the p-GaSe and n-Si provide a linear *I*–*V* relationship, we can conclude that the nonlinear rectifying nature of the *I*–*V* curve from the GaSe/Si p–n heterojunction in Fig. 4c must originate from the GaSe/Si heterojunction itself. The semi-log plot of the same *I*–*V* characteristics is shown in the inset of Fig. 4c. The device presents a very good rectifying characteristic, with a high forward current of nearly 100 μA at +5 V and a low-leakage current of nearly 0.1 μA at −5 V, offering a high current rectification ratio of nearly 1000.

The *I*–*V* characteristics of this vertical GaSe/Si heterojunction under dark conditions could be described by the thermionic emission model, given by eqn (1), which takes into account the current transport of majority charge carriers from the GaSe film to Si.^46^1
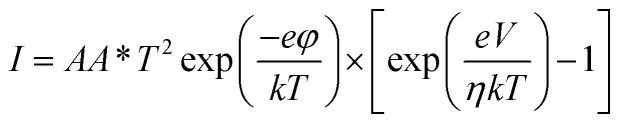
where *A* is the effective device area (∼22 μm^2^), A* is the effective Richardson-constant, which has a value of 112 A cm^−2^ K^−2^ for n-type Si, *φ* is the barrier height, *k* is the Boltzmann constant, and *η* is the ideality factor.

Moreover, the values of the ideality factor, *η*, and the barrier height, *φ*, can be evaluated with the help of the following equations:2
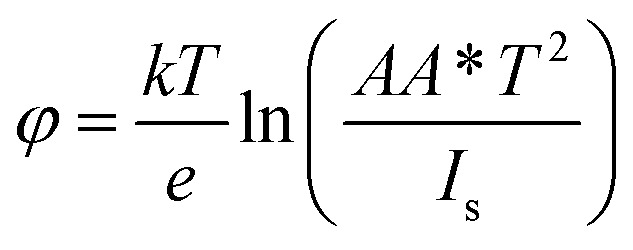
where *I*_s_ is the saturation current and is given by3
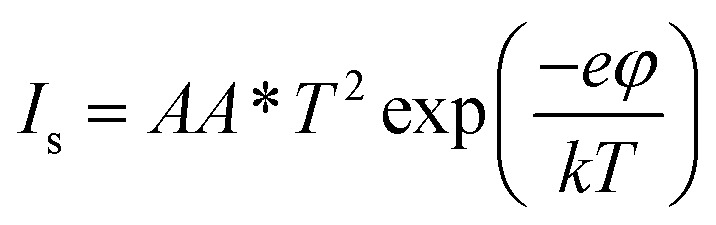
4
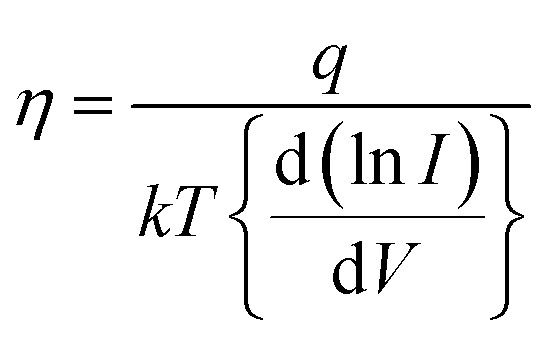


Based on the above equations, the values of the ideality factor and the barrier height have been estimated to be approximately 4.9 and 439 meV, respectively. This large ideality factor is attributed to the presence of interface states at the GaSe/Si junction. Since the p–n heterojunction diode has been fabricated *via* mechanical exfoliation with the help of scotch tape, the interface between Si and GaSe is not free from these interface states. As a result, the mechanism of carrier transport is no longer followed solely by thermionic emission, but some additional transport mechanisms such as recombination and tunneling become dominant, thereby increasing the value of the ideality factor. This large ideality factor value was also reported previously by several groups.^20,31^

**Fig. 5:**
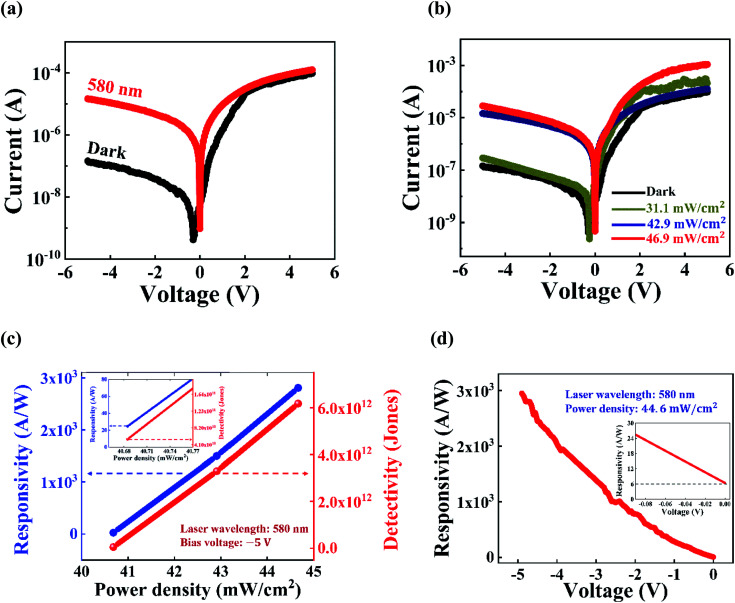
Semilog plot of the current–voltage characteristics of the as-fabricated device under (a) dark and illumination conditions (using a 580 nm laser with a power density of 46.9 mW cm^−2^), and (b) a fixed 580 nm laser light illumination with variable power densities. (c) The plots of the responsivity and specific detectivity with varying power densities. The inset shows the magnified plots of the same, highlighting the values of responsivity and specific detectivity at the lowest power density of 40.7 mW cm^−2^. (d) Variation of responsivity with applied bias voltage. The inset demonstrates the magnified plot of the responsivity *vs.* the applied bias curve near the zero bias voltage.

To study the photoresponse behavior of the as-fabricated p–n heterojunction diode, it was irradiated with a monochromatic light of wavelength 580 nm (energy: 2.1 eV, greater than the bandgap of GaSe). The light was vertically incident on the GaSe/Si heterostructure device, and the corresponding photo-generated current was patterned. A significant increment in current was seen compared to the dark current, which is clearly visible in Fig. 5a. The incident light generates adequate electron–hole pairs in the junction, which are promptly separated by the existing electric field, originating from the applied bias, and it contributes to the enhancement in current. A strong photoresponse was also observed even at zero bias, which clearly indicates the existence of a built-in potential in the heterojunction, thereby making it a promising candidate for self-powered photodetectors. To further investigate the photoresponsive effect at the GaSe/Si heterojunction, it was subjected to laser light of varying power densities ranging from 40.7 mW cm^−2^ to 44.6 mW cm^−2^, keeping the wavelength fixed at 580 nm, as illustrated in Fig. 5b. It was found that at an applied bias of −5 V and a fixed power density of 44.6 mW cm^−2^, the current in the p–n heterostructure device increases from −1.45 × 10^−7^ A to −3.21 × 10^−5^ A, which gives a photo-to-dark-current-ratio of about 221. In the reverse bias condition, the depletion width of the heterojunction increases, which results in significant enhancement of the electric field at the junction. The strong electric field decreases the carrier transit time, leading to reduced carrier recombination. Therefore, the large number of electron–hole pairs produced at the junction can be separated sufficiently and promptly, which is manifested as photocurrent. Upon increasing the power density of the incident illumination, a larger number of photo-generated charge carriers are available at the junction, thereby increasing the photocurrent to a greater extent. In contrast, under the forward bias condition, there exists a negligible depletion layer at the heterojunction, and hence the number of photo-generated charge carriers is relatively small as compared to the forward bias current. Therefore, because of the lack of availability of the photo-generated charge carriers in the forward bias, the photocurrent does not significantly increase the way it increases in the reverse bias condition upon increasing the incident power, as shown in Fig. 5b. A similar observation was reported by Lv *et al.*^16^ for their photodetector based on a graphene sandwiched GaSe/WS_2_ heterojunction. Shin *et al.*^26^ found a similar trend for their MoS_2_/Si vertical heterojunction photodetector.

The performance of the heterojunction photodetector was evaluated based on figure of merit parameters such as the photoresponsivity (*R*_*λ*_), noise equivalent power (NEP), specific detectivity (D*), and external quantum efficiency (EQE). Photoresponsivity quantifies the response of a photodetector when a photon is incident on it, which is calculated as the amount of photocurrent per unit incident power on the effective area of the device. It is mathematically represented by:5
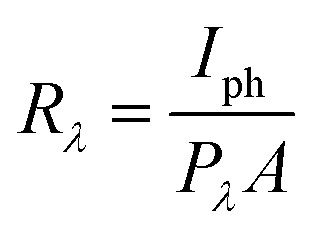
where *I*_ph_ is the photogenerated current (*I*_ph_ = *I*_illuminated_ − *I*_dark_), *P*_*λ*_ is the incident power density and *A* is the effective area of the device.

The specific detectivity gives a measure of the ability of a photodetector to distinguish weak optical signals from noise, and it is expressed as:6
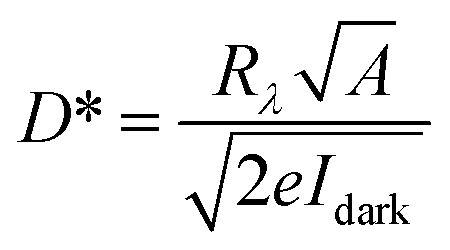
where *e* is the charge of an electron. The term in the denominator in eqn (6), 
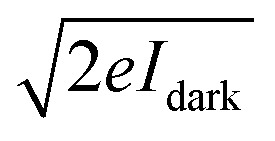
, represents the NEP, which expresses the minimum power detectable per square root bandwidth of a photodetector. The EQE measures the photon-to-electron conversion efficiency, and it is given by the following equation:7
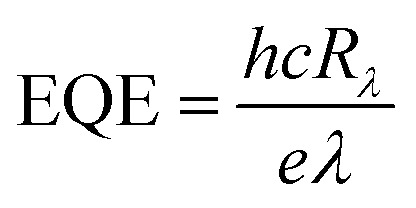
where *λ* is the wavelength of the incident light, *c* is the velocity of light in vacuum, and *h* is Planck's constant.

By using eqn (5) and (6), the variations of the photoresponsivity and the specific detectivity of the p–n heterojunction photodetector with power density were studied, which has been illustrated in Fig. 5c. As can be seen from Fig. 5c, there is a continuous rise in the values of both the photoresponsivity and specific detectivity with the power density of the incident illumination. The photocurrent increases significantly with the increasing power density, leading to high values of *R*_*λ*_ and *D*. For the present device, the effective device area is approximately 22 μm^2^. At a biasing of −5 V, with a 580 nm laser light of power density of 44.6 mW cm^−2^, the *D* and *R*_*λ*_ values of the photodetector have been evaluated to be ∼6.2 × 10^12^ Jones (1 Jones = 1 cm 
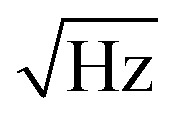
 W^−1^) and ∼2.8 × 10^3^ A W^−1^, respectively. The inset of Fig. 5c shows that under the minimum light illumination of 40.7 mW cm^−2^, both the responsivity and detectivity have significant values of approximately 25 A W^−1^ and 4.4 × 10^10^ Jones, respectively. These high values of *R*_*λ*_ and *D* point out that the GaSe/Si based photodetector is very sensitive to incident weak optical signals. The variation of *R*_*λ*_ with applied bias voltage was also studied, and it is plotted in Fig. 5d. As can be observed from Fig. 5d, *R*_*λ*_ increases with increasing bias voltage, and the inset of Fig. 5d clearly indicates that the device has an excellent photoresponsivity of 6 A W^−1^ even at zero bias. Under the zero bias, the electron–hole pairs generated from the incident illumination are swept in the opposite directions with the help of the built-in electric field present at the p–n heterojunction, giving a non-zero value of photoresponsivity; thus, the device is self-powered. Moreover, for the fabricated GaSe/Si p–n heterostructure, at a biasing of −5 V, the NEP value has been extracted to be 2.0 × 10^−13^ A Hz^−1/2^. Also, the EQE value was calculated by using eqn (7), and a high value of ∼6011 was obtained at a biasing of −5 V, which is very promising in the field of photodetection. A comparative study of the key parameters of a photodetector for our fabricated GaSe/Si p–n heterojunction device was carried out and is summarized in Table 1.

**Table 1:** A table of comparison of the important performance parameters of the photodetector in the present work and of previously reported photodetectors based on 2DLM/Si heterojunctions

Devices	Measurement conditions	Responsivity (A W^−1^)	Specific detectivity (Jones)	EQE	Response time	Reference
MoS_2_/Si	*λ* = 365 nm	7.2	∼10^9^	∼25	N.A.	30
	*V* = −2 V					
GaSe/MoS_2_	*λ* = 532 nm	0.35	∼10^10^	0.82	50 ms	47
	*V* = 2 V					
WSe_2_/GaSe	*λ* = 520 nm	6.2	∼10^10^	∼15	30 μs	48
	*V* = −1.5 V					
GaSe/WS_2_	*λ* = 410 nm	149	4.3 × 10^12^	∼450	37 μs	16
	*V* = 2 V					
GaSe/InSe	*λ* = 410 nm	350	3.7 × 10^12^	∼1060	2 μs	15
	*V* = 2 V					
GaSe/MoS_2_	*λ* = 520 nm	0.67	2.3 × 10^11^	1.6	155 μs	49
	*V* = 0 V					
WS_2_/Si	*λ* = 630 nm	1.15	∼10^11^	1.16	42 ms	50
	*V* = −1 V					
GaSe/Si	*λ* = 580 nm,	6	7.2 × 10^10^	12.85	1.4 s	This work
	*V* = 0 V					
	*λ* = 580 nm	2.8 × 10^3^	6.2 × 10^12^	6011		
	*V* = −5 V					

The excellent photoelectrical performance of our fabricated GaSe/Si-based 2D/3D vertical heterostructure device can be attributed to the following factors:

(i) The vertical structure of the device reduces the transport channel length of the photogenerated charge carriers, which reduces the probability of recombination and thereby effectively enhances the carrier collection efficiency.

(ii) The type-II band alignment and the presence of the built-in potential at the GaSe/Si p–n heterostructure aid efficient separation of the photogenerated charge carriers, leading to an increased lifetime of the photogenerated electron–hole pairs.

(iii) Generally, photodetectors exploiting the photovoltaic effect have poor responsivity due to the lack of internal gain.^51^ However, in this case, a photo-multiplication^52^ (PM) process comes into play when the heterojunction is illuminated with laser light under an applied bias. Trap states exist in GaSe and at the interface between GaSe and Si.^53^ These trap states prolong the lifetime of the photo-generated charge carriers so that the carriers can cycle the GaSe channel many times before recombination with the help of external bias.

In brief, the large electric field and the short transit time at the vertical 2D/3D heterojunction contribute significantly to increasing the photocurrent, which is directly manifested as high photoconductive gain, specific detectivity, and EQE, demonstrating its great potential towards future optoelectronic applications.

**Fig. 6:**
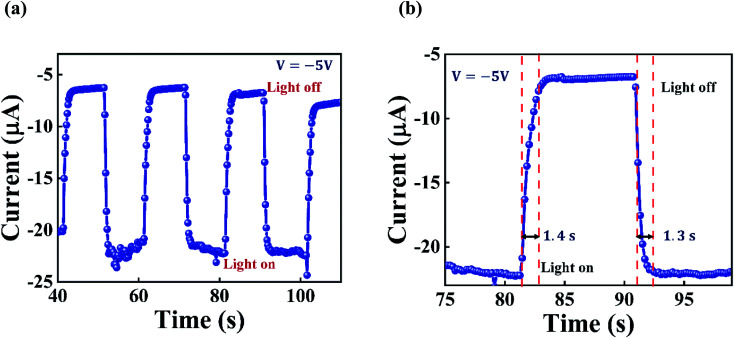
(a) Temporal response of the GaSe/Si p–n heterojunction device, observed under the illumination of a laser light with a wavelength of 580 nm and power density of 44.6 mW cm^−2^, at −5 V biasing. (b) Magnified curve of one response cycle.

The temporal response of the GaSe/Si vertical p–n heterojunction device was also recorded with the help of a 580 nm laser light, having a power density of 44.6 mW cm^−2^ at a constant bias voltage of −5 V. The laser light was illuminated on the device periodically by switching the laser between the on and off states with an interval of 10 seconds. As can be seen from Fig. 6a, the photocurrent increases sharply on the left edge of every pulse. This happens because of the sudden rise in photogenerated charge carriers upon illumination. Later, the photocurrent increases slowly and finally attains a constant value. A similar behavior is observed in the photocurrent when the laser light is switched off. Fig. 6b represents the magnified plot of one cycle, and it shows that the GaSe/Si p–n heterojunction-based photodetector has an approximate rise time of 1.4 seconds and decay time of 1.3 seconds. It is worthwhile to mention that the temporal response of the device was recorded by manual switching of the laser light without using any chopper. The slow response of the device can be accredited to the trap states present at the p–n heterojunction interface. Moreover, the GaSe flakes were mechanically exfoliated, and it has been reported that flakes obtained by mechanical exfoliation are prone to edge defects, which serve as traps to the photogenerated electron–hole pairs and reduce the overall speed of the fabricated device.^36,54,55^

**Fig. 7:**
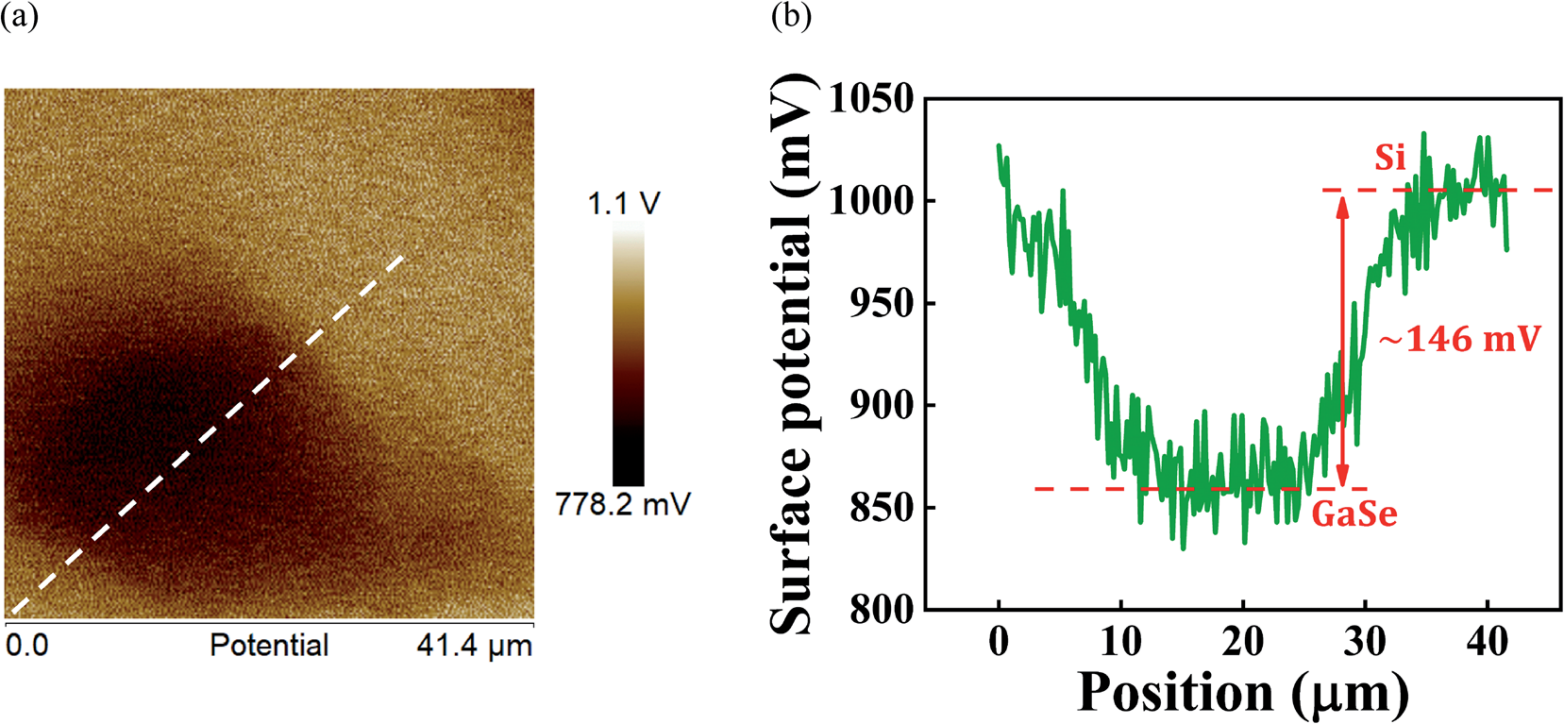
(a) Kelvin probe force microscopy (KPFM) image of the GaSe flake on Si substrate, indicating the change in surface potential between GaSe and Si. (b) Variation of surface potential with position across the GaSe/Si heterojunction along the dashed line depicted in (a).

We further carried out KPFM measurements on the vertical GaSe/Si p–n heterostructure to understand its band alignment. KPFM is one of the electrical modes of AFM; it is used to obtain workfunction values and information regarding the surface potential and charge at the nanoscale.^56^ A few research groups previously utilized this technique to examine the alignment of bands at the 2D/3D interface.^20,30^ KPFM basically measures the contact potential difference (CPD) between a conducting AFM tip and a sample surface by reading long-range electrostatic forces generated from the probe–sample interactions. In our present work, this technique was utilized to estimate the conduction band offset value at the GaSe/Si heterostructure. Fig. 7a portrays the surface potential mapping of the vertical GaSe/Si heterostructure, and Fig. 7b plots the variation of surface potential with respect to lateral distance at the GaSe/Si site along the dashed line as highlighted in Fig. 7a. The CPD between the sample substrate and the tip is defined as:8
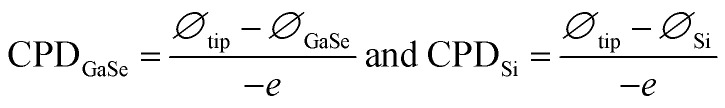
where *∅*_tip_, *∅*_GaSe_, and *∅*_Si_ are the work functions of the tip, GaSe, and Si, respectively.

The change in CPD values between GaSe and Si is given by9
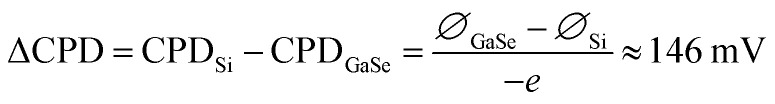


In Fig. 7b, this change in CPD between GaSe and Si at the GaSe/Si interface can be seen clearly. The surface potential value of the Si sample is 146 mV greater than that of the GaSe film. Therefore, the difference in work function values between Si and GaSe is evaluated as:

10 *∅*_Si_ − *∅*_GaSe_ ≈ 146 meV

This result, obtained from the KPFM measurement, gives direct proof of the junction formation between the GaSe flake and the Si substrate.

Next, to obtain the conduction band offset value for the heterojunction, we can use the following set of equations:

Considering the bandgap of GaSe as 2 eV,^57^ we have

11 *E*^GaSe^_C_ − *E*^GaSe^_V_ = 2.0 eV

Also, we know from the semiconductor fundamentals^58^ that12
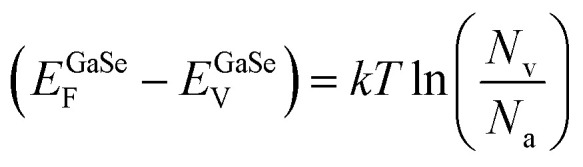


Subtracting eqn (12) from (11), we have13
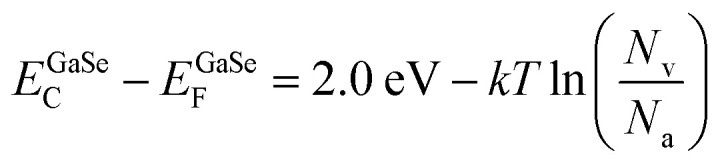


Also,14
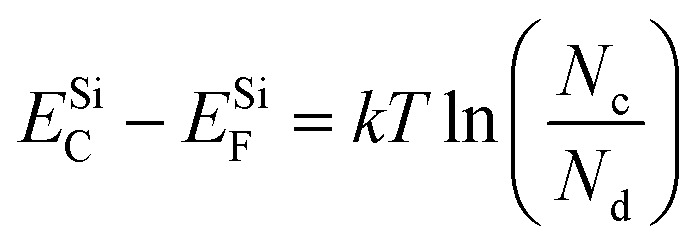
where *E*_C_, *E*_V_, and *E*_F_ are the energies corresponding to the conduction band minima, valence band minima, and Fermi level, respectively. *N*_v_, *N*_c_ are the effective density of states functions in the valence band and conduction band, respectively; *N*_a_ and *N*_d_ are the carrier densities of GaSe and Si, respectively. The values of *N*_v_ and *N*_c_ are given by 6.3 × 10^18^ cm^−3^ and 2.8 × 10^19^ cm^−3^, respectively. The carrier density of Si was found to be ∼10^16^ cm^−3^ by Hall measurements, and the carrier density of GaSe was taken to be ∼10^14^ cm^−3^.^59^ Substituting the values of *N*_v_, *N*_c_; *N*_a_, and *N*_d_ in eqn (13) and (14), we obtain

15 *E*^GaSe^_C_ − *E*^GaSe^_F_ = 1.714 eV

16 *E*^Si^_C_ − *E*^Si^_F_ = 0.205 eV

Subtracting eqn (16) from eqn (15), we can find the value of the conduction band offset, Δ*E*_C_, which is given by

17 Δ*E*_C_ = *E*^GaSe^_C_ − *E*^Si^_C_ = (*E*^GaSe^_F_ − *E*^Si^_F_) + 1.714 − 0.205

Therefore,

18 Δ*E*_C_ = 1.509 − (*E*^Si^_F_ − *E*^GaSe^_F_) = 1.509 − (*∅*_Si_ − *∅*_GaSe_)

The value of the workfunction difference, (*∅*_Si_ − *∅*_GaSe_), was experimentally found from KPFM measurements, as given in eqn (10). Thus, the conduction band offset value was calculated to be 1.36 eV.

As per Anderson's rule,^60^ the difference in electron affinity values between GaSe and Si provides the conduction band offset, Δ*E*_C_.

19 Δ*E*_C_ = *χ*_Si_ − *χ*_GaSe_

The reported values of *χ*_Si_ and *χ*_GaSe_ are 4.1 eV and 2.7 eV, respectively,^61^ which gives the value of Δ*E*_C_ to be 1.4 eV. This value is in good agreement with our experimentally obtained result.

**Fig. 8:**
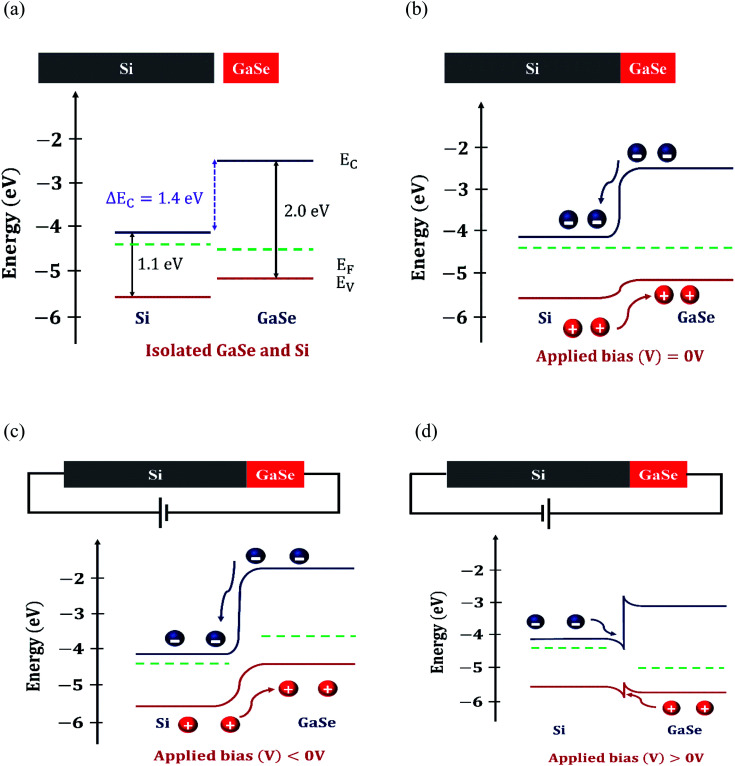
Band alignment at the vertical 2D/3D GaSe/Si heterojunction interface. (a) Band alignment for isolated GaSe and Si. The conduction band offset, Δ*E*_C_, has a value of ∼1.4 eV. (b–d) Band alignment at the GaSe/Si p–n heterojunction interface at different applied biases: (b) zero bias, (c) reverse bias, and (d) forward bias.

Further, the depletion width was estimated using the value of the built-in potential (*V*_bi_), and finally, the energy band diagram was drawn. The energy band diagram, shown in Fig. 8a–d, helps to understand the current transport in the GaSe/Si p–n heterojunction.

Ideally, the work function difference between GaSe and Si provides the built-in potential barrier.

20 *eV*_bi_ = *∅*_Si_ − *∅*_GaSe_ ≈ 146 meV as measured by KPFM

The depletion width is given by21
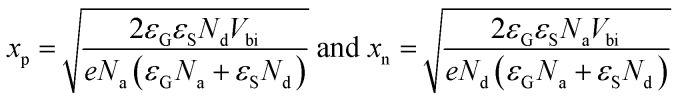
where *x*_p_ and *x*_n_ correspond to GaSe and Si respectively. *ε*_G_ and *ε*_S_ denote the dielectric constants of GaSe and Si, respectively. Putting these values in the above equations, the depletion widths in GaSe and Si were evaluated to be 132 nm and 1.32 nm, respectively.

Moreover, the built-in potential (*V*_bi_) values can be evaluated on both sides using the following equations:22
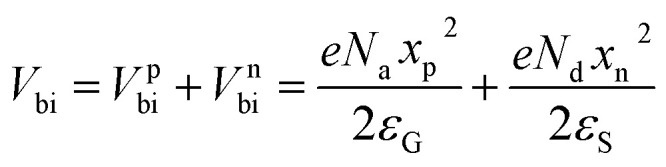

23 *V*^p^_bi_ = 144.7 mV and *V*^n^_bi_ = 0.74 mV

Therefore, the built-in potential barriers in the GaSe and Si sides were evaluated to be 144.7 mV and 0.75 mV, respectively. These values of the built-in potential and the depletion width are used to evolve the band diagram and charge transport.

To understand the current transport and photoelectrical behavior of the heterostructure device, a schematic energy band diagram, based on the type-II band alignment, has been drawn in Fig. 8. A conduction band offset value of approximately 1.4 eV exists in the heterojunction, as depicted in Fig. 8a. Under the zero bias condition, the Fermi levels of Si and GaSe become aligned, which causes band bending at the junction (Fig. 8b). Upon illumination, the photogenerated electron–hole pairs are collected by the electrodes with the help of the built-in potential present at the heterojunction, giving a non-zero photoresponsivity even at zero bias; thus, the device is self-powered. In Fig. 8c, when the heterostructure device is given negative biasing, a strong electric field is developed at the GaSe/Si heterojunction; as a result, the carrier transit time is shortened, which in turn reduces the carrier recombination. Thus, an excellent photoresponse is observed under this condition. Under the forward bias condition, as shown in Fig. 8d, the barrier height at the interface is reduced and a large amount of current flows through the junction.

## Conclusions

In this work, we have fabricated a vertical 2D/3D p–n heterojunction photodetector using mechanically exfoliated p-GaSe and n-type Si. The KPFM measurements on the device show that a type-II band alignment exists at the GaSe/Si heterojunction. Surface potential mapping at the heterojunction was carried out to extract the conduction band offset value, which was found to be ∼1.4 eV. The current–voltage measurements on the device reveal that the GaSe/Si photodiode exhibits a rectifying nature, with a high current rectification ratio of approximately 1000 at ±5 V. The photoresponse measurement on the fabricated device shows excellent performance, with high photoresponsivity, specific detectivity, and EQE values of ∼2.8 × 10^3^ A W^−1^, 6.2 × 10^12^ Jones, and 6011, respectively, at a biasing of −5 V. Moreover, an excellent photoresponse of 6 A W^−1^, obtained at the zero bias, suggests that the heterojunction device does not require any external power to operate. These results show the promising potential of self-driven GaSe/Si-based 2D/3D heterostructure devices in the field of photodetection. This work may pave the way to designing innovative optoelectronic devices by integrating low-dimensional materials with conventional 3D semiconducting materials.

## Author contributions

Sahin Sorifi: validation, formal analysis, investigation, data curation, visualization, writing – original draft. Shuchi Kaushik: methodology. Rajendra Singh: conceptualization, writing – review & editing, supervision, funding acquisition.

## Conflicts of interest

There are no conflicts to declare.

## Supplementary Material
